# Protection against the overuse and underuse of health care – methodological considerations for establishing prioritization criteria and recommendations in general practice

**DOI:** 10.1186/s12913-018-3569-9

**Published:** 2018-10-11

**Authors:** Cathleen Muche-Borowski, Dorit Abiry, Hans-Otto Wagner, Anne Barzel, Dagmar Lühmann, Günther Egidi, Thomas Kühlein, Martin Scherer

**Affiliations:** 1 Department of General Practice / Primary Care, University Medical Center Hamburg-Eppendorf (UKE), Martinistraße 52, 20246 Hamburg, Germany; 2 General Community medical practice, Bremen, Germany; 3 Institute for Primary Care, University Medical Center Erlangen, Erlangen, Germany

**Keywords:** Overuse, Underuse, Primary care, Guideline development, Clinical practice guidelines

## Abstract

**Background:**

Initiatives such as “Choosing Wisely” in the USA and “Smarter Medicine” in Switzerland have published lists of widely overused health care services. The German initiative “Choosing Wisely Together (Gemeinsam Klug Entscheiden)” follows this example. The goal of our study was to prioritize important recommendations against the overuse and underuse of health care services. The final list of recommendations will be published in the German guideline “Protection against the overuse and underuse of health care”.

**Methods:**

First, a multidisciplinary expert panel established a catalogue of prioritization criteria. Second, we extracted all the recommendations from evidence- and consensus-based German College of General Practice and Family Medicine (DEGAM) guidelines and National Health Care Guidelines (NVL). Third, the recommendations were rated by two independent panels (general practitioners and other health care professionals involved/not involved in guideline development). The prioritization process was finalized in a consensus conference held by DEGAM’s Standing Guideline Committee (SLK).

**Results:**

Eleven prioritization criteria were established. A total of 782 recommendations were extracted and rated by 98 physicians and other health care professionals in a survey. In the voting process, more than 80% of the recommendations were eliminated. After the final consensus conference, twelve recommendations from DEGAM guidelines, nine DEGAM addenda and 17 NVL recommendations were chosen for inclusion in the guideline, for a total of 38 recommendations.

**Conclusion:**

The selection procedure proved helpful in identifying the highest priority recommendations with which to combat the overuse and underuse of health care services. To date, in Germany there has been no attempt to compile such a list by using a systematic and transparent methodology. Hence, the guideline that results from this process can fill an important gap.

## Background

Internationally, there is growing awareness in the health care sector, politics, research and among the general public of the overuse and underuse of health care services. Among other examples, data confirm the prevalence of the excessive prescription of antibiotics [1, 2], overuse of cancer screening [3, 4], endoscopies [1], cardiac catheterizations [5, 6], hospital admissions [7] and aggressive end-of-life care [1]. Underuse of effective medical services can occur alongside overuse [8]. The failure to use effective medical interventions is documented worldwide with significant differences between and within countries including lack of access to and availability of services, clinicians’ poor adherence to evidence and guidelines, and patient non-adherence [8].

However, the extent of the overuse and underuse of health care services remains unclear due to the differing definitions of these terms, and the fundamental methodological problem that not all health care services have a generally accepted standard reference for appropriate usage (i.e. clinical practice guidelines) [1, 2, 9–11]. Without distinct concepts of appropriateness in health care, inappropriate care cannot be properly identified or measured. The indirect measurement of overuse and underuse based on regional differences in health care provision is less reliable than direct measurement and can only partially fill this gap [10–15]. Nevertheless, existing data depicting wide regional variations in health care provision within Germany may indicate the simultaneous presence of regional overuse and underuse of health care services in Germany [16].

Physicians’ associations are committed to the rational and moderate use of health care services, as can be seen in initiatives such as “Choosing Wisely” in the USA, “Smarter Medicine” in Switzerland and “Choosing Wisely Together (Gemeinsam Klug Entscheiden)” in Germany [17–19]. “Choosing Wisely” and “Smarter Medicine” have published lists of widely overused health care services and aim to steer doctor-patient communication towards this topic. These campaigns include a wide variety of methods for selection of appropriate health care services.

The discussion of excessive health care usage varies between countries. Compared to the aforementioned initiatives, a characteristic of the German discussion is the subordinate role of economic aspects. In the US initiative, “Choosing Wisely”, however, economic questions play a key role [2, 17]. Another feature of the German campaign is that both the excessive use of health care services and their underuse are addressed [18]. Considering overuse and underuse together gives credit to their interdependence: effective, low-cost interventions are sometimes neglected in favour of profitable, but less effective interventions [8].

Furthermore, the initiative for the protection against the overuse and underuse of health care services in Germany is affiliated with the German clinical guideline program [18, 20, 21]. This has a synergistic effect, since the strict requirements of high-quality guidelines regarding evidence, transparency and participation can be beneficial in the campaign against the excessive use and underuse of the health care system. In turn, clinical guidelines would benefit from an explicit declaration of which medical procedures are overused or underused [20].

The German umbrella organization of guideline development, the Association of Scientific Medical Societies in Germany (AWMF), therefore established the “Choosing Wisely Together” initiative in 2015. Under its supervision, the German College of General Practice and Family Medicine (DEGAM) is currently developing the evidence- and consensus-based guideline “Protection against the overuse and underuse of health care”, which comprises a precise list of recommendations for reducing excessive use and underuse in primary care in Germany.

The goal of this paper is (1) to introduce the methodology of the process used to identify and prioritize a concise number of top-priority recommendations for reducing the overuse and underuse of health care services from the perspective of general practitioners (GPs) in Germany and (2) to present the final list of selected recommendations.

## Methods

We developed a multi-step approach to prioritize guideline recommendations. Iterative rating rounds were held with various expert groups, taking existing recommendations from high-quality evidence- and consensus-based German guidelines as the basis. German guidelines were chosen as the basis because our target group are GPs practicing in Germany. Nevertheless, to receive a high-quality designation, German guidelines are formally obliged to integrate a systematic review of international evidence. The prioritization process was necessary for two reasons: first, the existing guideline recommendations are too numerous for GPs to feasibly utilize without further prioritization. Second, the prioritization process serves to select the most relevant recommendations for the protection against the overuse and underuse of health care services in primary care.

We established a structured process comprising the following seven steps:

1. Prioritization criteria

Delegates of the German Association of Internal Medicine (DGIM), the German Association of Epidemiology (DGEpi) and the German Network for Evidence-based Medicine (DNEbM) developed the prioritization criteria in an interdisciplinary nominal group process moderated by an AWMF representative. Criteria with at least 75% approval were accepted. The health care performance framework proposed by Arah et al. [22] was considered as well as the recommendations published by the German Institute for Applied Quality Promotion and Quality Assurance (aQua-Institute) for the development of quality indicators [23]. The participants of the nominal group process introduced further criteria. The resulting catalogue of agreed prioritization criteria was used to subsequently prioritize the guideline recommendations with the goal of reducing the overuse and underuse of health care services.

2. Extracting guideline recommendations

All recommendations were extracted verbatim from evidence- and consensus-based DEGAM guidelines and National Health Care Guidelines (NVL) and compiled in an Excel table. “Do” and “do not do” recommendations were likewise extracted, irrespective of the strength of the recommendations (“should”, “ought to”, “may be considered”). All up-to-date guidelines available in the AWMF guideline register as of July 2015 were included.

3. Rating panels

To account for the variation in perspective and characteristics between guideline developers and potential users of clinical practice guidelines (i.e. clinicians in the community), we established two different rating panels:

1. Guideline panel: Anyone involved in the development of the guidelines from which recommendations were extracted (authors, experts, patient representatives).
2. Naive panel: GPs not involved in the development of guidelines. All GPs affiliated with the Hamburg Department of General Practice/Primary Care were invited to join this group. The participants were randomly chosen from the list of GPs who had accepted the invitation. To assemble a clinically experienced panel, potential participants were required to prove that they treat at least 700 patients per quarter.

4. Rating

Both the guideline panel and the naive panel used the same rating procedure. All recommendations extracted from the guidelines were rated on a numeric rating scale from 1 (very low) to 9 (very high) according to the previously developed prioritization criteria. The members of the guideline panel rated only those recommendations from the DEGAM guidelines to which they themselves contributed. The naive panel rated all extracted recommendations (from DEGAM guidelines and NVLs). Given the high number of total extracted recommendations, voting for the naive panel was held in two consecutive rounds, one each for the DEGAM guideline and NVL recommendations, respectively. The panels were instructed to return questionnaires within eight weeks with several reminders issued. Participation in the naive panel was financially compensated (€ 600).

5. Algorithm-based data analysis

A multiple-step prioritization algorithm was developed based on the methodological recommendations made by the aQua-Institute for developing and evaluating quality indicators [19]. During this process the answers from both the guideline panel and the naive panel were combined. Descriptive statistics were used to analyze the data of both groups, and inductive statistics were implemented to evaluate the data from the naive panel only. This approach was chosen due to differences in size and composition between the groups.

Recommendations were selected from both panels if rated as high (7–9 points on the numeric rating scale) regarding the criterion “Relevance for overuse of health care” and/or “Relevance for underuse of health care” by at least 75% of the rating panel. To obtain an overall score for each of the selected recommendations, arithmetic means were calculated across all eleven prioritization criteria. Recommendations were then ranked by score for each guideline.

The number of recommendations selected by the naive panel was reduced further by applying t-tests to the overall scores of the recommendations, thereby identifying and sorting out scores significantly different from the highest score within each guideline (level of significance 1%). To do this, for each guideline the second score was compared with the first score via a one-sided t-test. If no significant difference was found, the third score was t-tested against the first score. This procedure was continued until a significant difference was found, at which point all previously tested recommendations were kept. The final recommendation tested, whose score was significantly different from the highest score, was excluded. All remaining recommendations were sorted out without further t-testing.

6. Evaluation of the prioritized recommendations by clinically active authors of the guideline “Protection against the overuse and underuse of health care”

The rationale behind this step was to further reduce the number of selected recommendations as well as to match the discrepancies in the results of the two panels. To ensure a clinical perspective, our four clinically active authors (MS, TK, GE, HOW) rated the guideline recommendations that had been prioritized by the two rating panels. Two aspects were chosen as the key criteria: “Relevance for overuse of health care” and/or “Relevance for underuse of health care”. An average of seven points or more on the scale for either of the aforementioned criteria was considered to be general acceptance of the guideline recommendation by the clinically active guideline authors.

7. Final prioritization of key recommendations by the DEGAM’s Standing Guideline Committee (SLK)

Following established procedures for developing an evidence- and consensus-based guideline, DEGAM’s Standing Guideline Committee (SLK) (more than 70 members, mainly GPs) was asked to complete the final prioritization procedure prior to publishing the recommendations in the guideline “Protection against overuse and underuse of health care”. Votes were cast on each of the guideline recommendations that had been prioritized and evaluated in steps 4–6, first in small moderated groups and after in a plenum. Two of these structured consensus conferences were held. The main question was: “Does the DEGAM wish to change the current utilization of this aspect of health care?” A recommendation was accepted if 75% or more participants agreed on the question in the plenum. The SLK was authorized to suggest amendments to the recommendations extracted from the full texts of the guidelines as well as DEGAM addenda (dissenting opinions of the DEGAM regarding guidelines from other specialties). Amendments and addenda were accepted if agreement was reached by 75% or more participants in the plenum. Recommendations upon which no voting took place in the plenum were integrated into a subsequent online survey.

## Results

1. Prioritization criteria

A multidisciplinary group comprising twelve members in total, namely five from DEGAM, one each from the DGIM, DGEpi, and DNEbM, and two methodologists, formally consented to the following eleven criteria under the moderation of two AWMF representatives:

1. *Clarity of the recommendation*
2. *Relevance of the recommendation for reducing the overuse of health care*
3. *Relevance of the recommendation for reducing the underuse of health care*
4. *Extent to which the respective misuse can be influenced*
5. *Feasibility of the recommendation*
6. *Quality of evidence*
7. *Strength of the recommendation*

*Relevance to health care provision goals:*

8) *Clinical goals*
9) *Public health goals*
10) *Further social goals*
11) *Relevance regarding patient safety*

2. Extracting guideline recommendations

Two authors (CMB, DA) independently extracted 782 recommendations from five DEGAM guidelines and nine NVLs. Column 3 of Table 1 indicates the number of recommendations that were taken from each DEGAM guideline and NVL.

**Table 1:** Number of guideline recommendations extracted and prioritized by guideline panel and naive panel

	Clinical guideline	Extracted recommendations	Recommendations prioritized by guideline panel and naive panel
DEGAM	Cough	16	11
	Chest pain	24	9
	Sore throat	58	11
	Fatigue	34	16
	Rhinosinusitis	15	1
	Total	147	48
NVL	Diabetes – treatment	39	5
	Diabetic neuropathy	120	5
	Diabetic nephropathy	63	4
	Diabetic foot	40	11
	Diabetes – patient education	32	3
	COPD	39	6
	Asthma	93	7
	Back pain	93	11
	Chronic congestive heart failure	116	25
	Total	635	77

*DEGAM-LL* DEGAM guideline, *NVL* National Health Care Guideline

3. Rating panels

In the guideline panel, an average of 17 guideline authors per DEGAM guideline were asked to contribute. The response rate averaged 37%. No patient representatives agreed to participate. More men (74%) than women (26%) took part. In total, 24 GPs (63%), nine representatives of other medical specialties (24%) and five representatives of other health professions (13%) participated.

**Fig. 1:**
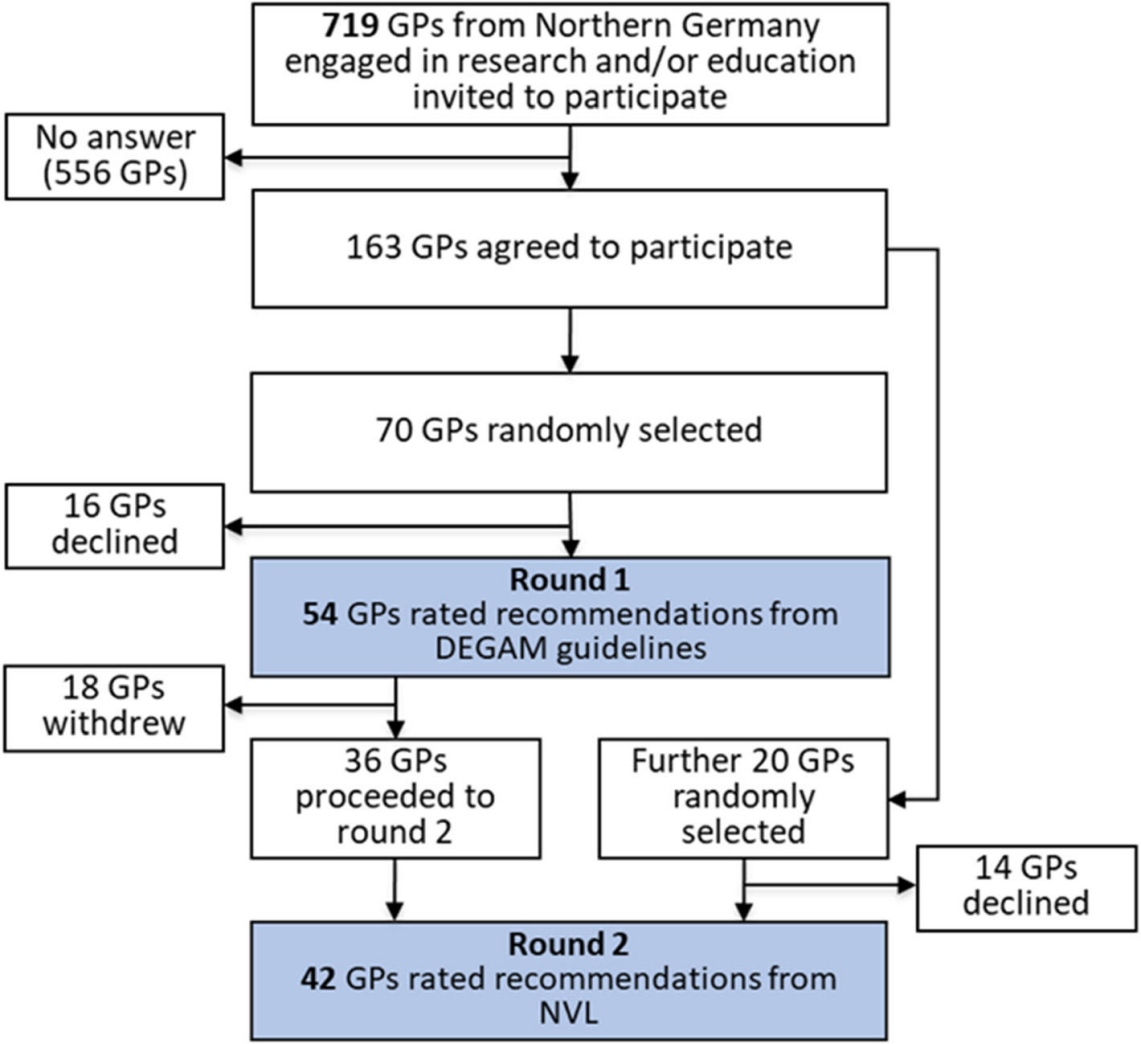
Flow of participants in the naive panel. GPs: general practitioners; DEGAM: German College of General Practice and Family Medicine; NVL: National Health Care Guidelines

The process of recruiting the naive panel is shown in Fig. 1. A total of 719 GPs were asked to participate. Of these, 163 agreed to participate, resulting in a response rate of 23%. Out of this group, we drew a random sample of 70 GPs, 54 of whom took part in the first round of rating, which focused on the recommendations from DEGAM guidelines (response rate 77%).

After re-recruiting before the second round of rating, this time concentrating on NVLs, 42 of the 74 recruited GPs took part (response rate 57%).

Approximately two-thirds of the participants in the naive panel were men (64%). Approximately 72% of the participants were GPs, and 20% were specialized in internal medicine. The number of physicians working in group practices (47%) and those working in a single-handed practice (43%) were evenly distributed. The average quantity of clinical experience was 23 years (+/− 8 years SD), 41% of the practices were located in larger cities, 26% in towns and surrounding areas, and 22% in rural areas. The group composition was similar in both rounds.

4. / 5. Rating and algorithm-based data analysis

A total of 125 guideline recommendations were rated as high priority. This accounts for 48 out of all 147 (33%) DEGAM recommendations and 77 out of all 635 (12%) NVL recommendations. Column 4 of Table 1 shows the number of prioritized recommendations per guideline. Of the 48 prioritized recommendations from the DEGAM guidelines twelve were selected by both panels, six solely by the naive panel and 30 solely by the guideline panel. As previously mentioned, the prioritization of NVL recommendations was based purely on the evaluation by the naive panel.

6. Evaluation of the prioritized recommendations by clinically active authors of the guideline “Protection against the overuse and underuse of health care”

**Fig. 2:**
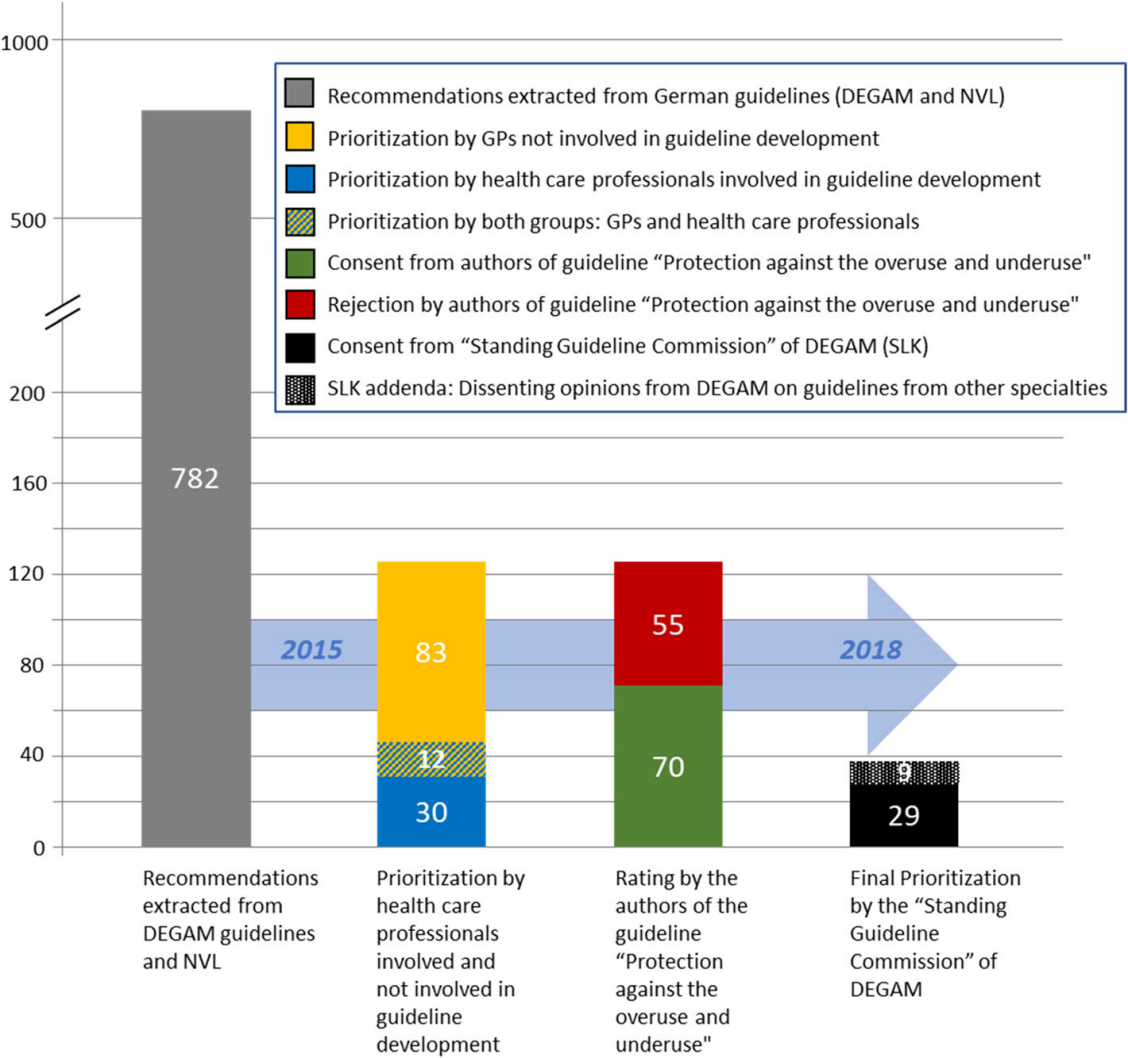
Prioritization process for the guideline “Protection against the overuse and underuse of health care”. (Methodological steps 4–7). 782 initially extracted guideline recommendations were condensed into 38 key recommendations. DEGAM: German College of General Practice and Family Medicine; NVL: National Health Care Guideline; GPs: general practitioners; SLK: DEGAM Standing Guideline Committee

The four clinically active authors (MS, TK, GE, HOW) of the guideline “Protection against overuse and underuse of health care” rated 33 of the 48 selected recommendations from the DEGAM guidelines as positive and 15 as negative. Of the 77 NVL recommendations 37 were considered positive and 41 negative (see Fig. 2).

7. Final prioritization of key recommendations by DEGAM’s Standing Guideline Committee (SLK)

Of the roughly 70 members of the SLK, 15 were involved in the first structured consensus conference on the guideline “Protection against the overuse and underuse of health care”; 34 participated in the second conference.

In the subsequent online survey, 21 of the 34 participants of the second conference took part (response rate 62%). The online survey addressed 37 recommendations. The final prioritization comprised 38 key recommendations (see Fig. 2). Of these 38 key recommendations, twelve were derived from DEGAM guidelines, 17 from NVL guidelines and 9 from DEGAM addenda.

A concise list of these key recommendations (21 for the protection against overuse, 16 against underuse and one recommendation protecting against both) is provided in Table 2, which reveals that the identified recommendations cover the entire span of primary care, from prevention and risk factor reduction (three recommendations), screening (three recommendations), diagnostics (eleven recommendations) and therapy (15 recommendations) to long-term care (six recommendations) (see Table 2). These categories are also frequently used as structural elements in guideline development processes.

**Table 2:** List of prioritized guideline recommendations for protection against the overuse and underuse of health care

Clinical guideline		Recommendation (short)	Protection against…^a^	Reference
Prevention/risk factor reduction				
Cough	1	Recommendation for regular documentation of smoking status in smokers with cough	Underuse	[38]
Asthma	2	Recommendation for prevention of passive smoking	Underuse	[39]
Back pain	3	Recommendation for physical activity in the prevention of back pain and back pain related sick leave	Underuse	[40]
Screening				
Skin cancer prevention	4	Recommendation against general skin cancer screening	Overuse	[42]
Alcohol-related disorders	5	Recommendation against general screening for at-risk drinking in primary care setting	Overuse	[43]
Prostate cancer	6	Recommendation against general PSA screening. Recommendation for a discussion of the pros and cons in patients asking for advice	Overuse	[44]
Diagnostics				
Sore throat	7	Recommendation for Centor Score and McIsaac Score to determine when to perform a throat swab	Overuse	[45]
Fatigue	8	Recommendation for two-question depression screening in patients with unspecific fatigue	Underuse	[41]
	9	Recommendation for basic diagnostics in every patient with fatigue (anamnesis, physical examination, blood work)	Underuse	[41]
	10	Recommendation against cancer diagnostics in patients with fatigue and no further signs of cancer	Overuse	[41]
Chest pain	11	Recommendation for Marburg Heart Score for estimating the probability of ischemic heart disease in patients with chest pain in the primary care setting	Overuse and Underuse	[46]
	12	Recommendation on coronary angiography only if defined diagnostic or therapeutic benefit is present	Overuse	[46]
Diabetic neuropathy	13	Recommendation on comprehensive diabetic foot examination	Underuse	[47]
Chronic congestive heart failure	14	Recommendation for echocardiography in every patient with congestive heart failure	Underuse	[48]
	15	Recommendation for echocardiography reports including an interpretation of the result	Underuse	[48]
Dementia	16	Recommendation for limiting imaging in dementia patients to those with possibly treatable disease	Overuse	[49]
Back pain	17	Recommendation against imaging in uncomplicated acute back pain	Overuse	[40]
Therapy				
Sore throat	18	Recommendation for patient-doctor discussion of the inappropriateness of antibiotics against viral infections	Overuse	[45]
Cough	19	Recommendation on rational antibiotic treatment of CAP	Overuse	[38]
	20	Recommendation against antibiotics in acute uncomplicated bronchitis	Overuse	[38]
	21	Recommendation against regular use of neuraminidase inhibitors in seasonal influenza	Overuse	[38]
	22	Recommendation against expectorants in acute infectious cough	Overuse	[38]
Diabetes – treatment	23	Recommendation for discontinuing diabetes medication when therapeutic goal is reached with minimal drug dose	Overuse	[50]
	24	Recommendation for rational HbA1c targets in patients with type 2 diabetes	Overuse	[50]
Diabetic foot	25	Recommendation on correct management of diabetic foot ulcers	Underuse	[51]
Chronic congestive heart failure	26	Recommendation against inappropriate medicines in patients with chronic congestive heart failure	Overuse	[48]
	27	Recommendation for ACE inhibitors in patients with reduced ejection fraction	Underuse	[48]
	28	Recommendation for beta blockers in patients with congestive heart failure	Underuse	[48]
Dementia	29	Recommendation for stopping drug treatment for dementia if dementia is exacerbated	Overuse	[49]
Back pain	30	Recommendation against IV or IM medications in unspecific acute or chronic back pain	Overuse	[40]
	31	Recommendation against IV NSAIDs in back pain	Overuse	[40]
Chronic ischemic heart disease	32	Recommendation for statin therapy without dose adjustment	Overuse	[52]
Long-term care in primary care setting				
Diabetes – treatment	33	Recommendation against regular monitoring of the lipid response to statin therapy	Overuse	[50]
Chronic congestive heart failure	34	Recommendation for appropriate management of elevated creatinine in patients with chronic congestive heart failure	Underuse	[48]
	35	Recommendation on content of regular control examinations	Underuse	[48]
	36	Recommendation for encouraging physical exercise in patients with stable congestive heart failure	Underuse	[48]
	37	Recommendation for weight monitoring in patients with congestive heart failure	Underuse	[48]
Dementia	38	Recommendation for additional focus on well-being of family members of a patient with dementia in primary care setting	Underuse	[49]

^a^Based on the mean scores of the prioritization criteria “Relevance for overuse of health care” and “Relevance for underuse of health care” assigned by the clinically active authors of the guideline “Protection against the overuse and underuse of health care”. A difference larger than one point between the means of the two criteria determined the category (protection against overuse vs. underuse). If the difference was one point or smaller, the recommendation was regarded as relevant for both overuse and underuse

## Discussion

### Major findings

The goal of this paper was to describe in detail the methodology of the prioritization process and to present the final list of prioritized recommendations for reducing the overuse and underuse of health care services in primary care in Germany. Our project resulted in a concrete and concise list of recommendations from DEGAM and NVLs forming the core of the soon-to-be-published DEGAM guideline “Protection against the overuse and underuse of health care”. In a multi-step prioritization process, 38 key recommendations were identified from a total of 782 extracted guideline recommendations.

The identified recommendations, which can be found in Table 2, cover the entire span of primary care. We would like to draw attention to two subsets of the prioritized recommendations, namely those regarding the topics of rational pharmacotherapy and recommendations relating to diagnostic aids.

With 14 out of 38 recommendations the largest portion of prioritized recommendations focused on rational pharmacotherapy. Three of these discussed the correct use of antibiotics. The inappropriate use of antibiotics is one of the best-documented examples of medication overuse – a global problem that has serious implications for antimicrobial resistance [1]. Two common examples of incorrect antibiotic use are viral upper respiratory tract infections (URTIs) and acute cough. Three recommendations selected in our prioritization process reflect an attempt to reduce this specific practice. One recommendation places emphasis on the dialogue between doctors and patients so that information is shared about viral URTIs and the appropriate use of antibiotics. Another recommendation advises against the use of antibiotics in uncomplicated acute bronchitis. The third recommendation advocates rational timing and the best choice of antibiotic therapy in community-acquired pneumonia (CAP).

Diagnostic aids represent practical tools for primary care practice with many implications for reducing the overuse and underuse of health care services. Therefore, it is the opinion of the authors that the diagnostic aids identified in this study merit more attention from GPs. Evidence-based diagnostic aids are intended to facilitate and standardize the diagnostic process and to identify patient subgroups who are more likely than others to benefit from further diagnostic procedures or treatment. This in turn helps to decrease both the overuse and the underuse of health care services. On the one hand, diagnostic aids lend structure to patient work-up, helping to ensure that no medical needs are overlooked. On the other hand, diagnostic aids can act as a barrier against so-called indication creep, i.e., the trend towards delivering a medical service to an increasing number of patients who do not belong to the population that clearly benefits from the service [24]. Furthermore, diagnostic aids promote patient-centered care by facilitating shared decision-making between patients and clinicians and increasing transparency. The informed patient represents a crucial factor in the reduction of overuse [25]. Our prioritization process identified three diagnostic aids: The Marburg Heart Score for risk stratification of coronary artery disease in patients presenting with chest pain in primary care, the two-question depression screening method, and the Centor Score and McIsaac Score for predicting GAS pharyngitis [26–30].

### Comparison with other initiatives

Our prioritized list of recommendations and the top-lists of “Choosing Wisely” (American Academy of Family Physicians) and “Smarter Medicine” (Switzerland) coincide in three areas: (1) imaging in uncomplicated low back pain, (2) antibiotic use in viral upper respiratory tract infections, and (3) PSA-screening.

Similarly to our study, the US initiative, “Choosing Wisely”, also aims to reduce overuse in health care as well as promote conversations between clinicians and patients about unnecessary medical services [17]. Having started with “top-5 lists” as a national campaign, “Choosing Wisely” has spread to more than 20 countries worldwide [31]. The methodological requirements for creating the “top-5 lists” include the documentation of the evidence-based process used to select and prioritize recommendations.

The initiative’s popularity has grown significantly due to the broad impact of public relations work, as well as through the publication of patient-friendly versions of the top-5 lists. Within just a few years, “Choosing Wisely” spread beyond the borders of the United States and now has branches in over 20 other countries (as of 2018), including Canada, Great Britain, Australia, Brazil, and the Netherlands [31, 32].

The prioritization process in “Smarter Medicine” (Switzerland) also presents an interesting methodological strategy for creating lists of measures with which to combat overuse in health care. It is based on evidence (literature search) that was subjected to a formal consensus-building process (Delphi technique). Additionally, four prioritization criteria are clearly identified: *general agreement on the recommendation; how often GPs are faced with the measure in question; direct costs; patient safety* [33]. However, the central methodological step in creating these lists was based on the opinions of only a few experts.

Unlike “Choosing Wisely” and “Smarter Medicine” the German campaign considers overuse and underuse simultaneously. The combined targeting of overuse and underuse acknowledges their interdependence: underuse is often related to competitive tensions between profitable vs. low-cost interventions, regardless of respective effectiveness [8]. Addressing underuse and overuse simultaneously enables our study to demonstrate to a skeptical public that patients’ interests are at the center of this initiative, not cost-cutting – a demand Howard Brody made in 2009 [34]. Moreover, the avoidance of overuse and underuse of health care services is a main objective according to the DEGAM resolution of 2002 [35].

Compared to the AWMF umbrella initiative “Choosing Wisely Together”, our guideline titled “Protection against the overuse and underuse of health care” introduces new prioritization criteria such as *relevance to health care service goals (in the sense of clinical goals or public health goals) and relevance regarding patient safety*. Overlapping prioritization criteria are also included, such as *the clarity of the recommendation, and references to overuse, underuse or misuse of the health care system.*

### Strengths and weaknesses of the methodological approach

As previously mentioned, the published data on the overuse and underuse of health care services is limited. This explains why our compilation of recommendations for the prevention of overuse and underuse mainly relied on consensus rather than an evidence-based process.

Our chosen method for developing the guideline “Protection against the overuse and underuse of health care” differs from classical guideline development (forming a representative committee, systematically searching for the best available evidence on the clinically relevant topic, critical evaluation of the relevant evidence, and formal consensus building concerning the recommended steps to be taken) [21]. As such, our decision to prioritize guideline recommendations that are already published has several benefits. High-quality guidelines published in the AWMF register in Germany are developed in a multidisciplinary group including patient representatives, are based on systematic searches for evidence, and are finalized in a formal consensus-finding process.

Our process of prioritizing existing guideline recommendations with the goal of reducing the overuse and underuse of health care services supplements the existing guidelines such that a similar prioritization has not yet been regularly included in the development of German guidelines. The prioritization emphasizes those guideline recommendations which we believe must be discussed by physicians and patients in a shared decision-making process. These goals are also pursued by the AWMF initiative “Choosing Wisely Together” [20]. Many recommendations in guidelines on individual issues in health care do not appropriately address the problem of the overuse and underuse of health care services appropriately, as proven by our selection process in which only 5% of the existing recommendations were prioritized.

Thus, the authors see this project as having the following strengths:

- Supplementation of current guidelines and their recommendations developed on an interdisciplinary basis
- Recommendations were extracted from systematically reviewed evidence
- The original wording of the recommendations was retained.

The fact that the naive panel consisted only of GPs affiliated with the Department of General Practice/Primary Care of the University Medical Center Hamburg-Eppendorf may limit our study’s generalizability to the entire population of GPs practicing in Germany. Nevertheless, the characteristics of the participants in this group were largely representative of the Germany-wide characteristics of GPs regarding age, gender, specialty and practice type [36]. A disadvantage of the rating process voiced by members of the naive panel was that the process was too time-consuming. This perception may have negatively influenced the accuracy and quality of answers given by the naive panel.

The NVL guideline groups were not surveyed concerning the NVL recommendations. This limitation is due to an agreement made between DEGAM, AWMF and DGIM. The DGIM, as an umbrella organization with its twelve associated sub-specialty societies, is largely represented in the development of NVLs but has devised its own “Choosing Wisely” recommendations which are currently in progress or in parts already published [37].

All DEGAM guidelines and interdisciplinary guidelines in which the DEGAM was involved undergo evaluation by the DEGAM’s Standing Guideline Committee (SLK) before publication. The Committee is an elected group of experienced clinically active physicians, GPs who have specialized in research, and residents in family medicine. All voice a common interest in working on and with guidelines for general practice.

The involvement of the SLK and face-to-face discussions in two conferences proved beneficial, as effective work took place in small, moderated groups with the results then presented in a plenum. This process is similar to that of a structured consensus conference. The task of the SLK members was to merge the different prioritizations made by the two panels. Thus, the SLK played a key role in ultimately prioritizing the key recommendations.

The executive board of DEGAM will authorize the final guideline in the same way other international initiatives such as “Choosing Wisely” or “Smarter Medicine” were authorized by their respective executive boards [17, 32].

The goal of this project was to present a concise evidence- and consensus-based list of recommendations by utilizing several innovative techniques: (1) the development of prioritization criteria in an interdisciplinary nominal group process, (2) the review of existing recommendations from high-quality evidence- and consensus-based German guidelines via iterative rating rounds with various rating panels, and (3) the use of a multi-step prioritization algorithm. Thereby, our project introduces new methodological impulses to the AWMF Guidance Manual and Rules for Guideline Development [21].

## Conclusion

Thus far, primary care has lacked a concise, clear, prioritized summary of the most important guideline recommendations aimed at reducing the overuse and underuse of health care services. We present the first German guideline that offers a concise list of recommendations for GPs as a supplement to the disease-related evidence- and consensus-based guidelines, therefore closing this gap. We integrated the guideline-user perspective (i.e., practicing GPs), thus presumably achieving greater likelihood of identification with and adherence to the recommendations among GPs. In addition, we intend for the list to promote shared decision-making between patients and physicians and effectively reduce the overuse and underuse of health care services. Future studies needed include evaluations of the success of the implementation of these key recommendations as well as the full guideline. This will further establish a valid basis for future health care research into the topic of overuse and underuse of medical services.

## Abbreviations

aQua-Institute: German Institute for Applied Quality Promotion and Quality Assurance
AWMF: Association of Scientific Medical Societies in Germany
CAP: Community-acquired pneumonia
COPD: Chronic obstructive pulmonary disease
DEGAM: German College of General Practice and Family Medicine
DGEpi: German Association of Epidemiology
DGIM: German Association of Internal Medicine
DNEbM: German Network for Evidence-based Medicine
GAS: Group A streptococcus
GP: General practitioner
NVL: National health care guideline
SLK: Standing guideline committee
URTI: Upper respiratory tract infections
